# Unveiling diagnostic challenges and therapeutic triumphs: endovascular management of a paediatric internal carotid artery pseudoaneurysm—a case report

**DOI:** 10.1093/ehjcr/ytaf372

**Published:** 2025-08-12

**Authors:** Zubair Farooq, Sachin Gautam, Abhinav Aggarwal, Preeti Gupta

**Affiliations:** Department of Cardiology, Vardhman Mahavir Medical College & Safdarjung Hospital, Ansari Nagar, New Delhi 110029, India; Department of Cardiology, Vardhman Mahavir Medical College & Safdarjung Hospital, Ansari Nagar, New Delhi 110029, India; Department of Cardiology, Vardhman Mahavir Medical College & Safdarjung Hospital, Ansari Nagar, New Delhi 110029, India; Department of Cardiology, Vardhman Mahavir Medical College & Safdarjung Hospital, Ansari Nagar, New Delhi 110029, India

**Keywords:** Internal carotid artery aneurysm, Endovascular coiling, Stent graft, Pediatrics, Case report

## Abstract

**Background:**

Internal carotid artery (ICA) pseudoaneurysms (PSAs) are exceedingly rare in the paediatric population and may arise secondary to infections, trauma, or congenital vessel wall abnormalities. Their diagnosis can be challenging due to non-specific presentations and a lack of established guidelines in children. Early recognition and appropriate intervention are critical, as complications such as rupture can be life-threatening. This case adds to the limited literature on paediatric ICA PSAs and highlights the role of endovascular treatment.

**Case summary:**

A 5-year-old boy presented with a pulsatile neck swelling persisting for 3 months, intermittent fever, and an episode of oral bleeding. Physical examination revealed a globular, pulsatile neck mass below the angle of the mandible, and imaging confirmed a right ICA PSA with a surrounding haematoma. A possible infectious or traumatic aetiology was considered. Due to the risk of rupture during intubation, a tracheostomy was performed to secure the airway. The PSA was successfully treated with endovascular coiling while preserving collateral circulation. Post-procedure recovery required intensive care monitoring due to intraoperative complications. However, the child was discharged with no neurological deficits, and at 1-month follow-up, the neck swelling had markedly reduced.

**Discussion:**

This case emphasizes the importance of considering both infectious and traumatic aetiologies in paediatric vascular lesions. It highlights the need for a multidisciplinary approach, tailored airway management, and individualized therapeutic strategies to achieve successful outcomes.

Learning points

- Paediatric internal carotid artery aneurysms are rare and can mimic infectious or traumatic conditions, requiring a high index of clinical suspicion.
- Endovascular coiling is a viable option in children, especially when long-term stenting poses risks due to vessel growth.
- A multidisciplinary, individualized approach with meticulous airway planning is essential, particularly when aneurysmal mass effect threatens airway integrity.

## Introduction

Cerebral aneurysms in the paediatric population are rare, comprising ∼0.5%–4.6% of all aneurysms diagnosed across all age groups.^1^ Among these, internal carotid artery (ICA) aneurysms are even less common and present unique diagnostic and therapeutic challenges. Unlike adult aneurysms, which are typically saccular and associated with atherosclerotic risk factors, paediatric aneurysms often demonstrate fusiform morphology and are more frequently linked to non-atherosclerotic causes such as congenital vascular anomalies, trauma, infection, and systemic inflammatory conditions.^2,3^

Infectious aneurysms, or mycotic aneurysms, may arise from direct bacterial invasion of the arterial wall, often secondary to upper respiratory infections or septic emboli, leading to inflammatory degradation of the vessel integrity. Recurrent tonsillitis, though common in children, has rarely been implicated as a precipitating factor in ICA aneurysm formation, though the anatomical proximity to cervical vessels may contribute to localized inflammatory spread.^4^ Traumatic pseudoaneurysms (PSAs), conversely, result from partial disruption of the arterial wall due to blunt or penetrating neck trauma, commonly involving the extracranial segment of the ICA.^5^

While both infectious and traumatic mechanisms are independently recognized causes of ICA aneurysms, their coexistence may have a synergistic effect. Inflammatory weakening of the arterial wall may predispose it to trauma-induced injury, or vice versa, where trauma creates a nidus for secondary bacterial colonisation. This interplay can obscure the primary aetiology, complicating both diagnosis and management.

We report the case of a 5-year-old boy who developed a right ICA PSA following recurrent tonsillitis and a suspected history of cervical trauma. This case illustrates the diagnostic complexity posed by overlapping infectious and traumatic factors in paediatric vascular pathology.

## Summary figure

**Figure:**
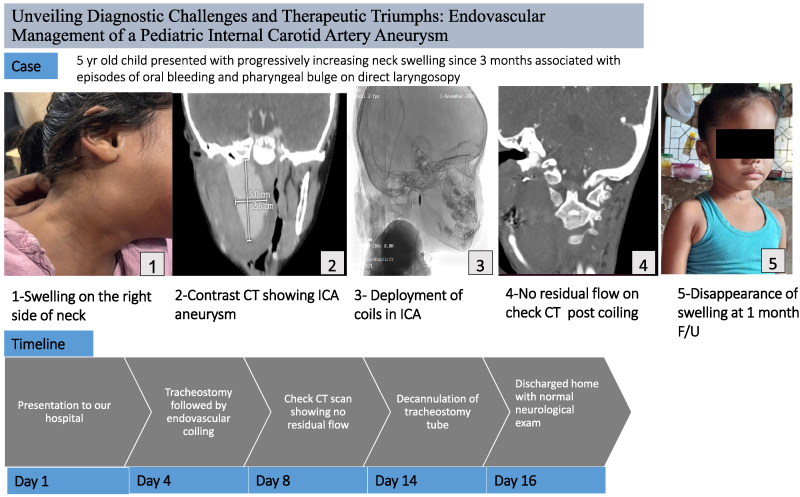


## Case presentation

**Figure 1:**
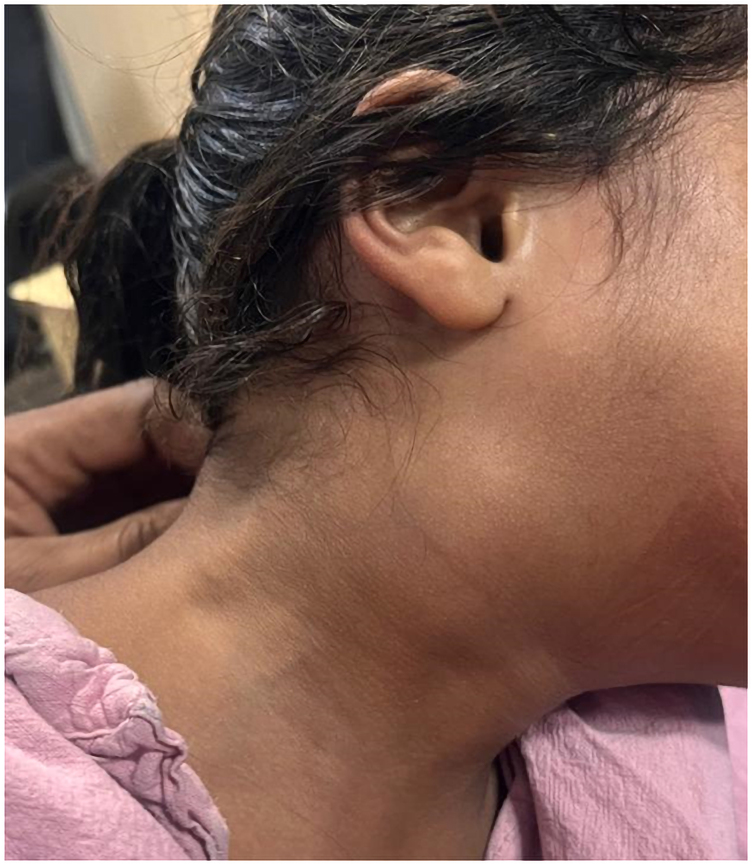
Clinical photograph showing a globular, pulsatile swelling on the right side of the neck, located below the angle of the mandible.

**Figure 2:**
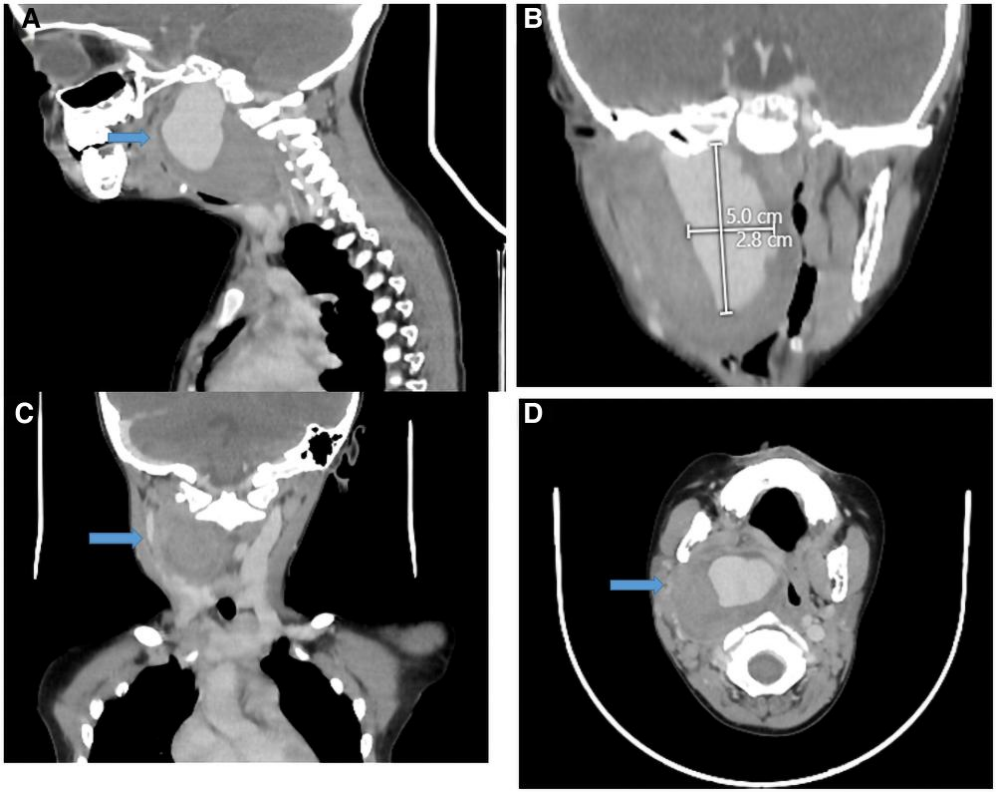
Contrast-enhanced CT scan of the neck demonstrating a pseudoaneurysm of the right internal carotid artery with surrounding parapharyngeal haematoma.

**Figure 3:**
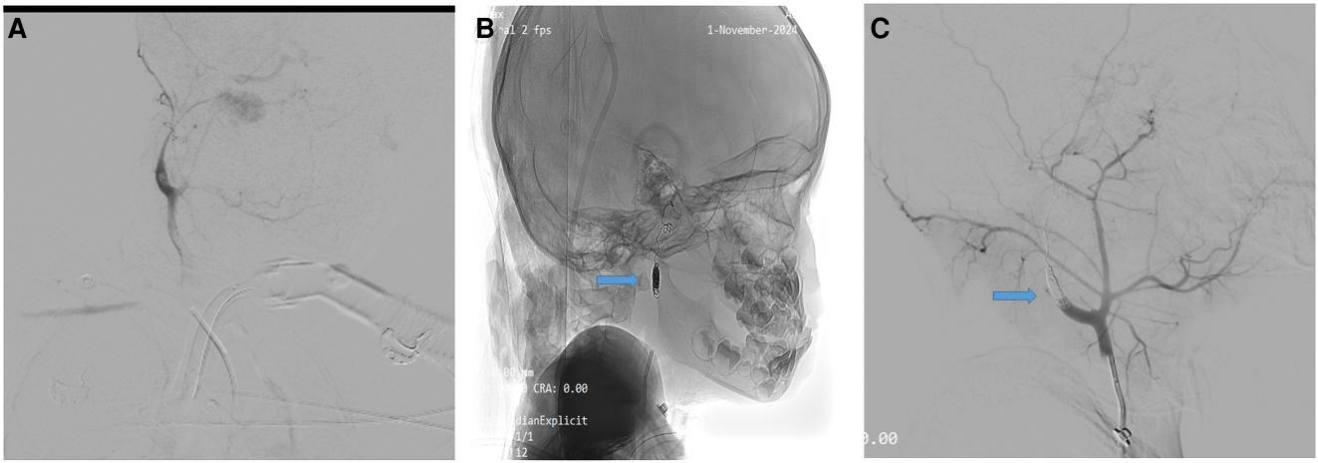
(*A*): Digital subtraction angiography showing the right ICA pseudoaneurysm arising from the cervical segment. (*B*): Intraoperative image demonstrating coil deployment within the aneurysmal sac using the Axium Prime Detachable Coil System (Medtronic). (*C*): Post-procedure angiographic image confirming complete occlusion of the pseudoaneurysm and right ICA segment, with robust collateral cross-flow.

The patient was a 5-year-old boy presenting to the hospital with a history of a progressively enlarging neck swelling, intermittent fever, and a recent episode of oral bleeding. The child had no known history of chronic illnesses, systemic infections, or genetic disorders. His medical history included recurrent episodes of acute tonsillitis treated with multiple courses of antibiotics. There was a history of a minor neck injury while playing with schoolmates ∼4 months before presentation. The family history was unremarkable for vascular or connective tissue disorders, and there were no significant psychosocial stressors reported. The patient had been treated conservatively for tonsillitis with antibiotics and antipyretics for 3 months, with partial symptomatic relief. On physical examination, the patient appeared anaemic and had a pear-sized, globular, pulsatile swelling located on the right side of the neck below the angle of the mandible [*Figure 1*]. Systemic examination was unremarkable, with no neurological deficits or other abnormalities noted. Relevant laboratory investigations revealed mild anaemia (haemoglobin: 9.3 g/dL; normal range: 11.5–15.5 g/dL), a mildly elevated white blood cell count (10.6 × 10³ cells/mm³; normal range: 4.5–11 × 10³ cells/mm³), and a slightly elevated platelet count (565 × 10³ cells/mm³; normal range: 150–450 × 10³ cells/mm³). [*Table 1*]. These findings indicated a low-grade inflammatory or reactive process. Differential diagnoses considered were mycotic aneurysm secondary to recurrent tonsillitis, traumatic PSA due to a neck injury, or other vascular anomalies such as arteriovenous malformations, which may present similarly in paediatric patients and require differentiation through advanced imaging modalities like magnetic resonance (MR) angiography. Imaging confirmed a PSA of the right ICA with an adjacent haematoma in the parapharyngeal space [*Figure 2*]. A multidisciplinary team reviewed the case, emphasising the risks of rupture during intubation. A direct laryngoscopy revealed a bulge in the lateral pharyngeal wall. A tracheostomy with a cuffed tube of 6 mm was performed to secure the airway, and endovascular coiling was planned. Under general anaesthesia, right femoral arterial access was obtained with a 5Fr sheath. Diagnostic imaging of the left vertebral artery, left common carotid artery, and right common carotid artery showed a large pseudo-aneurysm measuring ∼3.4 × 3.3 mm (*Figure 3A*). Micro-catheter cannulation of the right ICA was performed, followed by deployment of four coils measuring 6 mm × 20 cm, 5 mm × 15 cm, 3 mm × 8 cm, and 4 mm × 12 cm [Axium prime detachable coil system, Medtronic] (*Figure 3B*). A post-deployment check shot showed complete obliteration of the right ICA from the petrous to cervical segments with no residual opacification of the aneurysmal sac (*Figure 3C*). Check imaging confirmed robust cross-flow from the left vertebral and left internal carotid arteries across the anterior and posterior communicating arteries (*Video 1*).

**Table 1:** Baseline investigations of the patient

Investigation	Value	Reference range	Units
Hb	9.3	11.5–17.0	g/dL
Total leukocyte count	10.6 × 10^3^	4.0–10.0 × 10^3^	Cells/mm^3^
Platelets	565 × 10^3^	150–500	Cells/mm^3^
Urea	14.3	17.0–43.0	mg/dL
Creatinine	0.36	0.60–1.30	mg/dL
AST	33	10–35	U/L
ALT	13	10–45	U/L
ALP	130	40–128	U/L
Bilirubin (total)	0.59	0.30–1.20	mg dL

**Figure 4:**
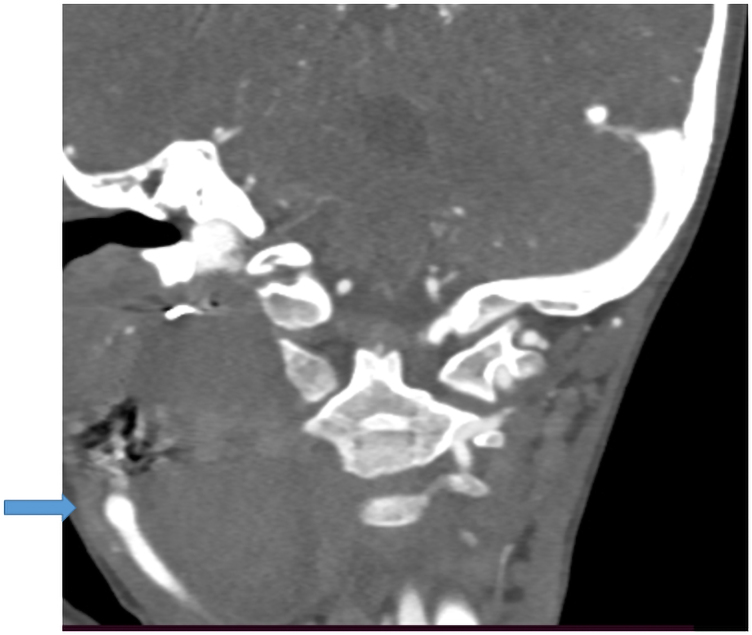
Postoperative CT angiogram (day 4) showing no residual aneurysmal filling and a resolving parapharyngeal haematoma.

**Figure 5:**
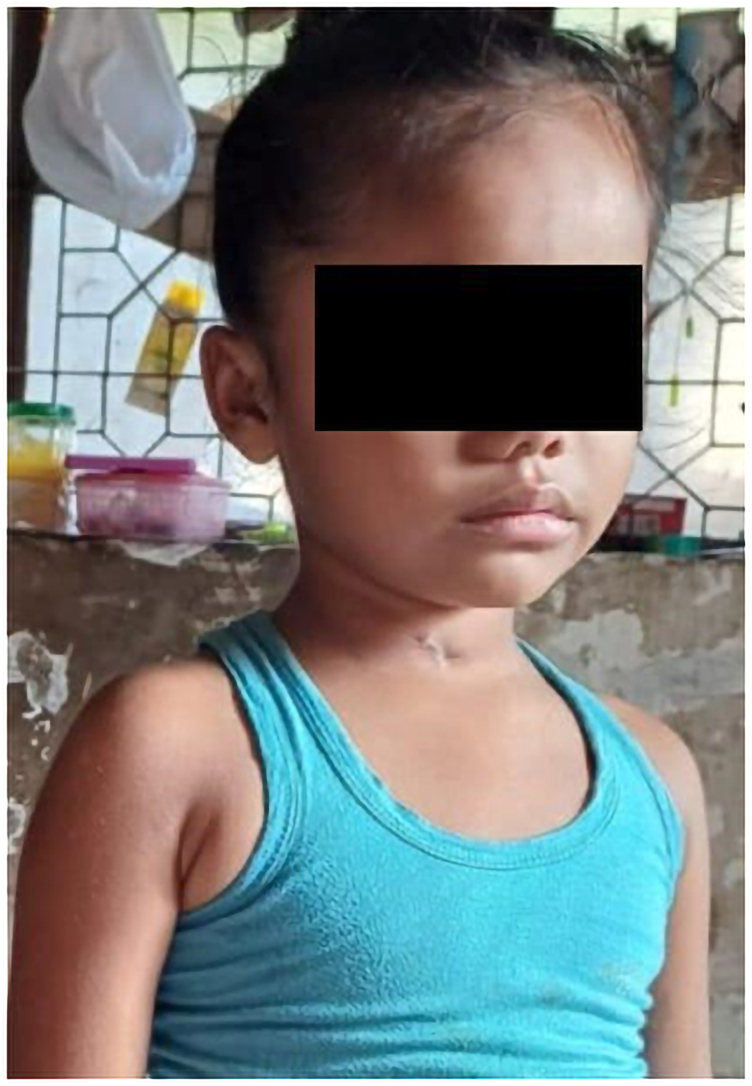
Clinical photograph 1-month post-repair showing disappearance of the neck swelling.

During the procedure, the child developed significant oral bleeding estimated at ∼200–250 mL, likely secondary to mucosal erosion overlying the aneurysmal segment, which was exacerbated by instrumentation during airway management. This was accompanied by hypoxia due to inadvertent right mainstem bronchial intubation through the tracheostomy tube, resulting in left lung collapse as evidenced by fluoroscopy. Aggressive fluid resuscitation with crystalloids and transfusion support was instituted, and airway repositioning was promptly performed. The tracheostomy tube was downsized from 6 to 5 mm to improve ventilation dynamics and avoid further trauma. These intraoperative complications highlight the inherent risks of airway and vascular manipulation in such anatomically constrained and inflamed regions. A follow-up CT angiogram on post-procedure day 4 showed no residual flow in the aneurysm sac (*Figure 4*). Following intensive care support for intraoperative complications, the child's postoperative course was carefully monitored. He demonstrated progressive clinical improvement and was discharged on day 16 after tracheostomy tube removal, with a normal neurological examination and partial resolution of the neck swelling. He was seen again at 1 month follow-up and showed a remarkable decrease in the size of the swelling (*Figure 5*).

## Discussion

This case describes a rare occurrence of a right ICA PSA in a 5-year-old child, presenting with neck swelling and intermittent fever. A PSA, or false aneurysm, arises when there is a breach in the arterial wall, typically involving disruption of the intima and media layers, with blood escaping into the surrounding tissue. Unlike a true aneurysm—which involves dilatation of all three layers of the arterial wall (intima, media, and adventitia)—a PSA is contained only by the adventitia or peri-vascular connective tissue. This results in the formation of a haematoma that remains in continuity with the arterial lumen.^14^ PSAs may develop following trauma, infection, iatrogenic injury, or inflammation, and are prone to rupture due to the lack of structural vessel wall integrity. In this case, both infection and trauma may have contributed to the formation of the PSA. The trauma, possibly from a previous injury, along with the inflammation caused by the acute tonsillitis, may have weakened the arterial wall and facilitated the development of the PSA.^6^ In children, infections of the head and neck, such as tonsillitis, can potentially lead to mycotic aneurysms through direct invasion of the vascular wall by pathogens.^4^ On the other hand, trauma is a known mechanism for aneurysm formation due to vessel wall injury, which can result in pseudo-aneurysm development.^5^ The diagnosis of ICA PSAs in children is challenging due to their rarity and non-specific symptoms. In this case, CT angiography of the neck was crucial in identifying the PSA, providing insights into its size, location, and the surrounding haematoma. Invasive angiography was essential in perioperative planning, allowing real-time evaluation of vascular anatomy and collateral flow, which was critical for deciding on the best management approach.^7^ While digital subtraction angiography remains the gold standard for vascular imaging, advanced non-invasive modalities such as MR angiography can offer high diagnostic accuracy without ionising radiation. This is particularly advantageous in paediatric patients, where repeated imaging may be necessary for monitoring lesion progression or treatment response.^8^ The management of paediatric ICA PSAs requires a multidisciplinary approach. Surgical intervention is often necessary given the risk of rupture and potential neurological complications.^9^ Endovascular techniques, such as coil embolisation and stent-graft placement, offer minimally invasive options. In this case, although stent-graft placement was initially considered, concerns about long-term patency, vessel growth, and the need for lifelong antiplatelet therapy along with technical difficulties in manoeuvring the stent to the desired area led to the decision to proceed with coil embolisation.^10^ Our approach allowed for PSA occlusion while preserving cerebral blood flow, given the adequate collateral circulation confirmed on angiography. Only a limited number of paediatric ICA PSA cases have been reported, with aetiology varying between mycotic, traumatic, and idiopathic. Most documented cases emphasize the utility of CT and magnetic resonance imaging for diagnosis, with treatment strategies largely determined by PSA location and collateral circulation adequacy. Treatment strategies in paediatric vascular cases must account for anatomical and physiological differences compared with adults, such as smaller vessel calibres, higher vessel elasticity, and ongoing somatic growth. These factors can affect device selection, procedural risks, and long-term outcomes, particularly with stents, which may not accommodate future vessel growth, making endovascular coiling a more favourable option in many paediatric cases.^11^ In cases with suspected mycotic origins, literature suggests aggressive management with antibiotics alongside surgical intervention. However, without a clear infectious origin, cases like this one highlight the need for adaptable management plans. In a similar case of an ICA PSA with a neck haematoma, the condition was managed with an endovascular stent-graft placement.^12^ In another study, two patients with ICA PSAs were successfully treated with endovascular coiling, which involved parent artery occlusion using coils. This technique resulted in good angiographic outcomes without any neurological complications, and no re-canalization of the aneurysms was observed on follow-up. They also conclude that endovascular coiling may be better option in children as they have good collateral flow.^13^ This case highlights critical procedural considerations, particularly regarding airway management in the presence of a large cervical PSA.^11^ The occurrence of significant intraoperative bleeding and hypoxia emphasized the need for meticulous airway planning, including real-time fluoroscopic guidance, pre-procedure tube size evaluation, and readiness for rapid tube repositioning. Future cases may benefit from preoperative simulation and involvement of paediatric anaesthesiologists experienced in complex airway scenarios to minimize risk during intubation or tracheostomy in vascularly compromised neck anatomy. This case report has certain limitations. First, the absence of histopathological or microbiological confirmation limits the ability to definitively establish an infectious aetiology for the PSA. Second, the lack of long-term follow-up data restricts the assessment of delayed complications, vessel remodelling, or aneurysm recurrence, which are critical in the paediatric population given their prolonged life expectancy and ongoing vascular development.

## Conclusion

Paediatric ICA PSAs are exceedingly rare and can present with overlapping features of infectious and traumatic aetiologies, necessitating a high index of clinical suspicion. Their management poses unique diagnostic and therapeutic challenges due to the involvement of critical anatomical structures. A multidisciplinary approach, with careful pre-procedural planning—including thorough airway assessment—is essential to mitigate procedural risks. Endovascular coiling offers a viable and less invasive treatment option in children, particularly in scenarios where long-term stent patency and somatic growth are considerations. Postoperative care must remain vigilant to potential complications, and clinical strategies should remain adaptable to intraoperative developments. Further research is needed to inform standardized guidelines for the diagnosis and management of these complex vascular lesions in children.

## Patient’s perspective

“When our little boy’s neck first started swelling, we honestly thought it was something that would go away with a few antibiotics. But it didn’t. The swelling just wouldn’t go down, and he kept getting fevers on and off. Then, when he had bleeding from his mouth just before we brought him in, we were terrified. As parents, seeing your child go through something like that is so hard—you feel helpless.

When we came to the hospital, the doctors explained everything carefully. We were shocked to learn he had an aneurysm, something we’d never even heard of in a child. And then there was this worry about how to treat it without putting him in more danger. The idea of him having a tracheostomy felt scary and overwhelming, but we also trusted the doctors and could see they were being extremely careful with every decision.

Going through his surgery was probably one of the hardest things we’ve experienced as a family. Every hour felt like forever. But after the procedure, seeing him recover, seeing his strength—it was amazing. Our little boy was so resilient, even when he didn’t fully understand what was happening.

Now that we’re home, we feel deeply grateful. Grateful to the doctors and nurses who took such incredible care of him and grateful for the simple things, like hearing him laugh and watching him play. This experience taught us just how strong he is, and as a family, we’ve learned to cherish every moment. We’ll always carry this experience with us, but now with a sense of relief and gratitude.”

## Data Availability

The data underlying this article cannot be shared publicly due to privacy concerns related to patient confidentiality. Further details can be made available from the corresponding author upon reasonable request, in compliance with institutional and ethical regulations.

## Lead author biography

Dr. Zubair Farooq is a dedicated cardiology fellow at Vardhman Mahavir Medical College & Safdarjung Hospital, New Delhi. He earned his MBBS from Sheri Kashmir Institute of Medical Sciences, Srinagar, Kashmir, followed by an MD in Pediatrics & Neonatology from the same institution. Currently pursuing a DM in Cardiology, Dr. Zubair is committed to furthering his expertise in cardiology, with a strong focus on clinical excellence and patient care.
